# Comparison of percutaneous nephrolithotripsy combined with retrograde intrarenal surgery and multi-tract percutaneous nephrolithotripsy for octopus stone: A propensity score-matching study

**DOI:** 10.1097/MD.0000000000038311

**Published:** 2024-05-31

**Authors:** Deheng Cui, Guoqiang Chen, Jianbin Luo, Qinghong Ma, Guangzhi Wang, Zesong Yang, Liefu Ye

**Affiliations:** a Department of Urology, The Second Hospital of Longyan, Longyan, Fujian, China; b Shengli Clinical Medical College, Fujian Medical University, Fuzhou, Fujian, China; c Department of Urology, Fujian Provincial Hospital, Fuzhou, Fujian, China.

**Keywords:** octopus stone, percutaneous nephrolithotripsy, retrograde intrarenal surgery, visual analogue scale

## Abstract

To compared the effectiveness and safety of single standard mini percutaneous nephrolithotripsy (SM-PCNL) combined with retrograde intrarenal surgery (RIRS) and multiple standard mini percutaneous nephrolithotomy (MSM-PCNL) in the treatment of octopus stone of 2 to 4 cm. The clinical data of SM-PCNL combined with RIRS and MSM-PCNL for octopus stone with a 2 to 4 cm diameter from October 2019 to December 2022 were analyzed retrospectively, and propensity score matching was used to screen patients. The matched patients were paired, and the operation time, complications, postoperative pain, tubeless rate, stone-free rate (SFR), and postoperative hospital stay were further compared between the 2 groups. 88 patients underwent SM-PCNL combined with RIRS (combined group), and 143 patients underwent MSM-PCNL (multiple channel group). After matching analysis, there were 49 patients in each group, and there was no significant difference in the general preoperative data between the 2 groups. The perioperative complications and stone-free rate were no statistical difference. In postoperative pain (4.00 ± 0.74 vs 5.00 ± 0.74, *P* = .00), tubeless rate (44.90% vs 20.41%, *P* = .01), hemoglobin drop (9.38 ± 7.48 vs 14.22 ± 7.69, *P* = .01), postoperative hospital stay (3.37 ± 1.09 vs 5.08 ± 1.29, *P* = .00), the combined group was significantly better than the multiple channel group. Regarding operation time, the combined group was more than the multiple channel group (103.27 ± 27.61 vs 78.39 ± 19.31, *P* = .000). For octopus stone with a diameter of 2 to 4 cm, the effectiveness and safety of SM-PCNL combined with RIRS were similar to those of MSM-PCNL The surgeon should carefully evaluate the patient’s physical condition, stone characteristics, and expectations before the operation and assist the patient in choosing an appropriate plan.

## 1. Introduction

Urinary calculus is a common human disease; the latest research show that the incidence of kidney stone in Chinese adults is as high as 5.8 %.^[1]^ For the large burden of kidney stone, the preferred surgical method is percutaneous nephrolithotomy (PCNL).^[2]^ It has been over half a century since Willard Goodwin first reported PCNL in 1955.^[3]^ The technology is becoming increasingly mature, but there are still some unavoidable complications, such as pain, fever, sepsis, septic shock, and bleeding, which may lead to nephrectomy and even life-threatening in severe cases.^[4]^

Staghorn stone are mostly branched, satellite-shaped, or extended into parallel calices. For kidney stone with a diameter >4 cm, establishing multiple channels in clinical to improve the stone-clearing efficiency and SFR is often necessary, which may cause more surgical trauma.^[5]^ Zeng et al pointed out that for kidney stone with a 2 to 4 cm diameter, microchannel PCNL could reduce postoperative pain, blood transfusion rate, and hospitalization days compared with standard percutaneous nephrolithotripsy (S-PCNL). However, the SFR between microchannel percutaneous nephrolithotomy and S-PCNL do not have difference.^[5]^ It was worth noted that in clinical practice, we used to encounter a particular type of staghorn stone, which was relatively difficult to manage. This type of stone is characterized by a small distribution in the main body of the stone at renal pelvis with a maximum diameter of 2 to 4 cm, while the branches extending into the renal calices are thin and long, resembling octopuses. To distinguish it from ordinary staghorn shaped stones, we named it “octopus stone.” These patients usually do not have hydronephrosis, and the sharp of calices are long and narrow. In PCNL operation, obtaining a better lithotripsy range through the swing of the nephroscope is difficult, especially for larger nephroscope. In addition, due to the relatively narrow neck of the renal calices, the swing of the nephroscope can tear the neck of the renal calices, causing uncontrollable bleeding. Thus, 2 or more channels are needed to achieve an excellent stone-clearing effect. Each center has no uniform treatment plan for this type of kidney stone. Our center mainly use single standard mini PCNL (SM-PCNL) combined with RIRS and multiple standard mini percutaneous nephrolithotomy (MSM-PCNL) to treat those patients. This study summarized and compared the effectiveness and safety of these 2 surgical methods, aiming to provide the basis for selecting clinical treatment schemes.

## 2. Materials and methods

The data of 231 patients with octopus stone (2 to 4 cm in diameter) treated in our center from October 2019 to December 2022 were analyzed retrospectively. The patients who received SM-PCNL combined with RIRS were defined as combined group, and those who received MSM-PCNL were described as multiple channel group. Exclusion criteria: patients with ipsilateral ureteral stricture, solitary kidney, ectopic kidney, horseshoe kidney, uncontrolled urinary tract infection, immune deficiency, uncontrollable hemorrhagic disease, severe cardiopulmonary insufficiency, renal tumor, and patients that unable to cooperate with surgical posture.

Both groups underwent complete blood count, routine biochemistry, coagulation test, urine routine, urine bacterial culture, kidney-ureter-bladder (KUB) X-rays, and computed tomography urography before the operation. For patients with positive urine bacterial culture before the operation, selected sensitive antibiotics according to the drug sensitivity results, and treated until the urine culture turns negative. For those with negative preoperative urine cultures, a single dose of antibiotics (second-generation cephalosporins or quinolones) was used to prevent infection before surgery. Patients with diabetes were given insulin therapy until the fasting blood glucose was controlled below 8 mmol/L. The general preoperative data of the 2 groups of patients were shown in Table 1.

**Table 1:** General information of the 2 groups (before PSM).

Variable	Combined group (n = 88)	Multiple channel group (n = 143)	*P* value
Age (years)	53.43 ± 9.99	51.52 ± 11.36	.20
*Gender, n* (%)			
Male	50 (56.81)	71 (49.65)	.34
Female	38 (43.18)	72 (50.35)	
BMI (kg/m^2^)	22.15 ± 4.07	23.57 ± 3.45	.01*
Creatinine (mg/dL)	52.72 ± 16.06	55.61 ± 12.60	.13
History of ESWL/URS	6 (6.82)	13 (9.09)	.63
*Comorbidities*			
None	74 (84.09)	127 (88.81)	.32
Hypertension/diabetes	14 (15.91)	16 (11.19)	
*Stone site, n* (%)			
Right	44 (50.00)	67 (46.85)	.685
Left	44 (50.00)	76 (53.15)	
Stone diameter (mm)	29.14 ± 5.87	26.06 ± 4.18	.00*
Urine culture n (%)	30 (34.09)	43 (48.86)	.56
Stone CT density (HU)	751.16 ± 224.13	551.15 ± 175.77	.00*

BMI = body mass index; CT = computed tomography; ESWL = extracorporeal shock wave lithotripsy; HU = hounsfield units; PSM =propropensity score-matching; URS = ureteroscopic lithotripsy.

*P < .05, statistically significant difference.

Non-contrast computed tomography (NCCT) images were performed on postoperative day 1 for the first assessment of postoperative stone residual. The patient’s second evaluation was at the end of the fourth postoperative week through KUB X-rays. A 5F double J stent was indwelling in all patients during the operation, and it was removed under local anesthesia after 4 weeks. Patients with residual stone may needed supplementary treatment. The same experienced urologist performed all procedures.

This study was approved by the Ethics Committee of the Second Hospital of Longyan City, Fujian Province, and informed consent was obtained from all patients (LYEYEC2023-019). The individual in this manuscript had given written informed consent to publish these case details. Our study was conducted in accordance with the ethical standards of the 1964 Declaration of Helsinki and its subsequent amendments.

## 3. Surgical approaches

### 3.1. Combined group

**Figure 1.**
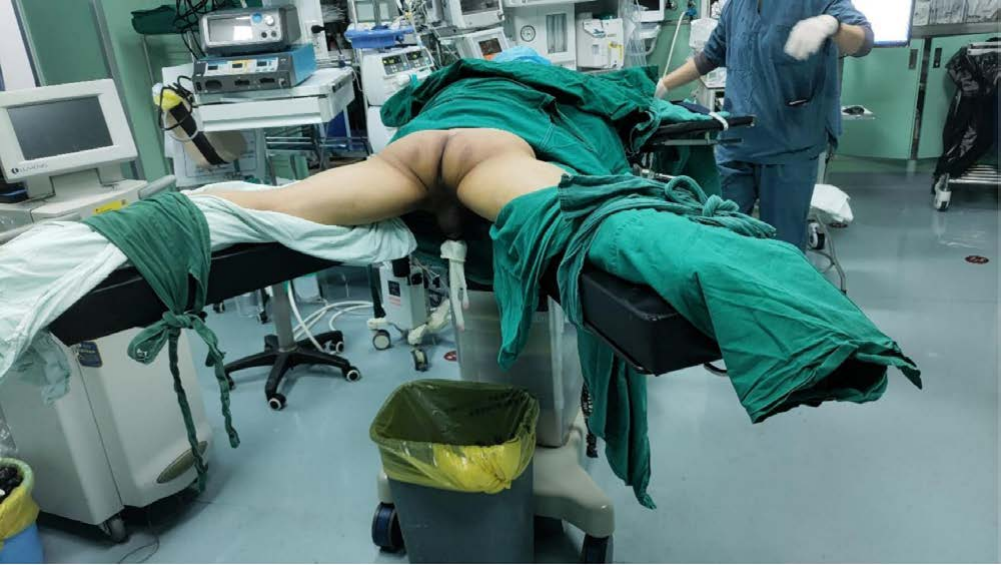
The patient was prone position, with both lower limbs straight and properly extended outward.

**Figure 2.**
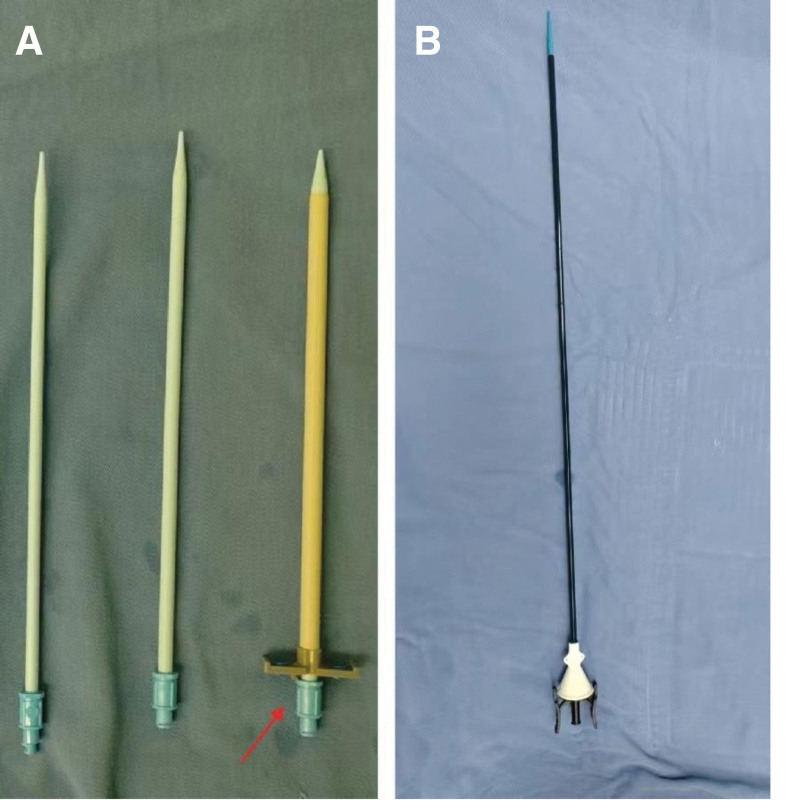
Percutaneous sheath of 18F (left) and ureteral access sheath of 12F (right).

All patients received general anesthesia with endotracheal intubation. First, the patient was placed in the lithotomy position, and a 5F ureteral catheter was indwelled into the ipsilateral ureter using a cystoscope. Then changed to a prone position (Fig. 1), and under the guidance of ultrasound, an 18-gauge Chiba needle was used to enter the kidney, and the 18F~20F working channel were established at middle or lower calices routinely (Fig. 2). A 12F nephroscope (R. WOLF Company, Knittlingen, Germany) was used to dust the stone. The height of the lavage fluid from the operating bed was kept at 80 to 100 cm. A COOK 0.38-inch guide wire (Cook Medical) was inserted through the ipsilateral ureteral catheter. A 12/14F ureteral access sheath (Cook Medical, Fig. 2) was pushed into the ureter through the guide wire, with the tip at the ureteropelvic junction. First, 550 μm optical fiber (Lumenis holmium laser) was used for lithotripsy through the nephroscope. The energy was set to 1.5 to 2.0 J, and the frequency was set to 20 to 30 Hz. When the stone in the visual field were removed, a 7.5F flexible ureteroscope (Karl Storz, New Delhi) was used to retrograde into the kidney through the ureteral access sheath to look for residual stones, and break the residual stones with 200 μm optical fiber (Lumenis holmium laser, 0.8–1.0 J and the 20–25 Hz). A nitinol basket (Cook Medical) was used to remove the fragments through the percutaneous renal channel. Routinely, nephrostomy tubes were not indwelled without pyonephrosis, stones combined pus moss, obvious bleeding or a second operation was planned. The blood routine was rechecked on the first day after the operation, and the nephrostomy tube was removed 2 to 3 days after the operation if placed.

### 3.2. Multiple channel group

The anesthesia procedures, body position, and indwelling ureteral catheter were the same as those of the combined group. Two suitable puncture points were found with ultrasound guidance, and fascial dilators were used to expand the channel to 18F to 20F gradually. The 550 μm holmium laser fiber was also used to dust the stone in the visual field as much as possible and the fragments were remove by circulating water flow through the channels. Then, ultrasound was used to examine the distribution of the remaining stones, and other channels (18F–20F) were established to remove the remaining stones. The standard of indwelling nephrostomy tube was the same as those of the combined group. The blood routine was rechecked on the first day after the operation, and the nephrostomy tube was removed 2 to 3 days after the operation if placed. A 5F double J stent was indwelling in all patients during the operation, and it was removed under local anesthesia after 4 weeks.

### 3.3. Study variables

**Figure 3.**
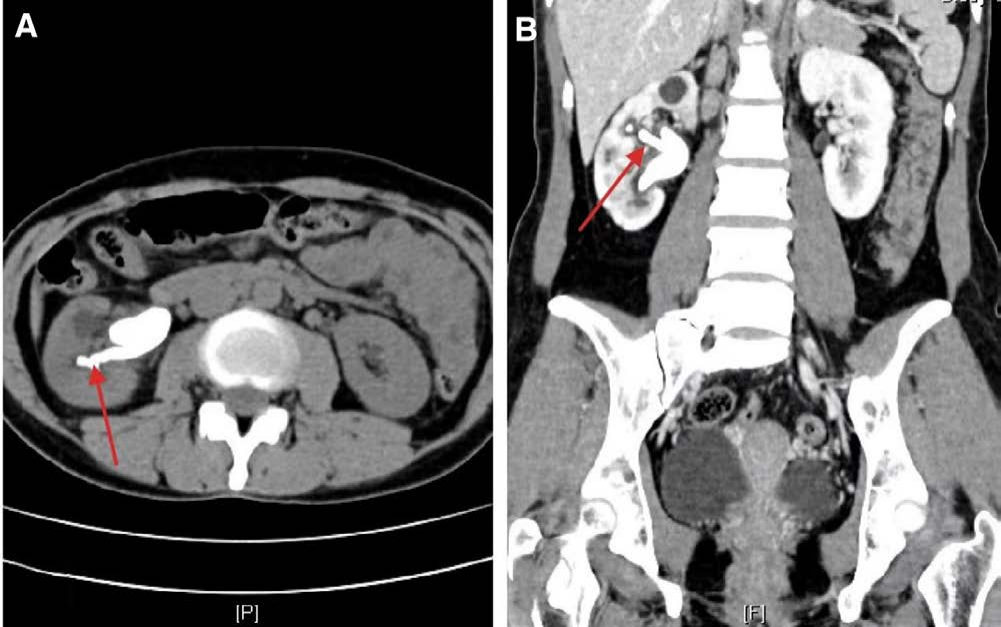
The red arrows in the transverse (A) and coronal (B) planes of CT show elongated branches of staghorn calculi. CT = computed tomography.

Octopus stone: ① The main body of the stone is located in the renal pelvis, ② has 2 or more thin and long branches that extend into the minor calyces of the kidney, ③ has a longest diameter of 2 to 4 cm (Fig. 3).

Visual analogue scale (VAS) was used for postoperative pain score; SFR: no residual stone or asymptomatic stone with a diameter <2 mm.^[6]^ Complications were recorded according to the modified Clavien classification. The operative time was calculated from the start of the percutaneous puncture to the completion of nephrostomy tube placement. The drop in hemoglobin was defined as the difference between the hemoglobin on the 1st day before surgery and the 1st day after surgery. The criterion for blood transfusion was that the hemoglobin level was <7 g/dL. The diameter of the calculus referred to the longest diameter measured on the plain film in the KUB area.

Postoperative infection was considered as one of the following manifestations: (1) positive blood culture, (2) positive urine culture, (3) body temperature > 38.5 °C that excluding fever caused by other systemic infections and other factors.^[7]^ According to the latest Sepsis-3 diagnostic criteria,^[8]^ sepsis is defined as life-threatening organ dysfunction caused by the host’s dysregulated response to infection, with a rapid increase in Quick SOFA score ≥ 2. Definition of septic shock: patients with sepsis undergo adequate treatment, but persistent hypotension persists after volume resuscitation, requiring vasoconstrictor drugs to maintain mean arterial pressure ≥ 65 mm Hg, and serum lactate level > 2mmol/L. All complications related to infection were recorded until 1 month after the operation.

### 3.4. Statistical analysis

All variables were presented as mean ± standard deviation or as numbers (%). Due to retrospective observational studies, there are many biases and confounding variables in the data. The propensity score matching (PSM) is precisely to reduce the impact of these biases and confounding variables and eliminate interference factors between groups. PSM analysis was performed using a multivariate logistic regression model based on: age, gender, body mass index (BMI), preoperative creatinine, history of extracorporeal shock wave lithotripsy/ureteroscopic lithotripsy, comorbidities (hypertension/diabetes), side of stone, stone diameter, positive preoperative urine culture, mean stone CT density (hounsfield units). Pairs of 49 patients were derived using 1:1 greedy nearest neighbor matching within a PS score of 0.02. After propensity score matching, Paired Student *t* test (for continuous variables) and the McNemar test (for classified variables) were used to analyzing the differences between the 2 groups of variables. *P* < .05 was considered to be statistically significant. All statistical analyzes were performed using commercially available software SPSS 23.0.

## 4. Results

After the screening, 231 patients were included in this study. Eighty-eight patients underwent SM-PCNL combined with RIRS, and 143 patients underwent MSM-PCNL (see Table S1, Supplemental Digital Content, http://links.lww.com/MD/M649, which illustrates details of patients in the 2 groups before propensity score-matching was performed). There was no significant difference between the 2 groups in mean age, gender, preoperative creatinine, history of extracorporeal shock wave lithotripsy/ureteroscopic lithotripsy, complications (hypertension/diabetes), side of stone, positive preoperative urine culture. The mean stone CT density (751.16 ± 224.13 vs 551.15 ± 175.77, *P* = .00) and stone diameter (29.14 ± 5.87 vs 26.06 ± 4.18, *P* = .00) of the patients in the combined group were larger, and the BMI (22.15 ± 4.07 vs 23.5 ± 3.45, *P* = .01) were lower than the multiple channel group. After propensity score matching, 49 patients in each group were included in the analysis, and there was no statistical difference in general characteristics between the 2 groups (Table 2). All subsequent statistical operations were performed between new subjects.

**Table 2:** General information of the 2 groups (after PSM).

Variable	Combined group (n = 49)	Multiple channel group (n = 49)	*P* value
Age (year)	54.22 ± 9.91	52.10 ± 12.33	.38
*Gender, n* (%)			
Male	29 (59.18)	28 (57.14)	1.00
Female	20 (40.82)	21 (42.86)	
BMI (kg/m^2^)	22.82 ± 4.44	22.34 ± 3.31	.58
Creatinine (mg/dL)	55.82 ± 18.11	52.49 ± 12.79	.31
History of ESWL/URS	2 (4.08)	5 (10.20)	.38
*Comorbidities*			
None	39 (79.59)	41 (83.67)	.79
Hypertension/diabetes	10 (20.41)	8 (16.33)	
*Stone site, n* (%)			
Right	21 (42.86)	22 (44.90)	1.000
Left	28 (57.14)	27 (55.10)	
Stone diameter (mm)	26.73 ± 4.92	26.96 ± 4.57	.82
Urine culture n (%)	17 (34.70)	17 (34.70)	1.00
Stone CT density (HU)	670.71 ± 174.01	666.88 ± 162.59	.88

ESWL = history of extracorporeal shock wave lithotripsy, PSM =propropensity score-matching, URS = ureteroscopic lithotripsy.

For patients after PSM, the postoperative supplementary treatment rate and stone-free rate of the 2 groups showed no statistical difference. The mean postoperative hospital day, mean hemoglobin drop, postoperative VAS, and tubeless rate of the combined group were all better than those of the multiple-channel group, with statistical differences (Table 3). On the contrary, the operation time of the combined group was significantly longer than multiple channel group (103.27 ± 27.61 vs 78.39 ± 19.31, *P* = .00). However, the 2 groups had no statistical difference in total postoperative complications, including fever, sepsis, septic shock, and blood transfusion rates (Table 4).

**Table 3:** Perioperative findings of 2 groups. RIRS, retrograde intrarenal surgery.

Variable	Matched sample		
	Combined group	Multiple channel group	*P*
Postoperative hospital day (days)	3.37 ± 1.09	5.08 ± 1.29	.00
Mean hemoglobin drop (g/L)	9.38 ± 7.48	14.22 ± 7.69	.01
Mean operative time (min)	103.27 ± 27.61	78.39 ± 19.31	.00
Tubeless rate, n (%)	22 (44.90)	10 (20.41)	.01
*Secondary intervention*			
None	42 (85.71)	43 (87.76)	1.00
ESWL/RIRS/second-look	7 (14.29)	6 (12.24)	

ESWL = history of extracorporeal shock wave lithotripsy.

**Table 4:** Perioperative complications and postoperative effect evaluation.

Variable	Matched sample		
	Combined group	Multiple channel group	*P*
Significant complication	16 (32.65)	25 (51.02)	.09
*Clavien grade I*			
Fever	3 (6.12)	4 (8.16)	1.00
Pain	3 (6.12)	5 (10.2)	.50
Hematuria	1 (2.04)	2 (4.08)	1.00
*Clavien grade II*			
Transfusion	1 (2.04)	3 (6.12)	.63
Urinary leakage	2 (4.08)	2 (4.08)	1.00
Perirenal hematoma	2 (4.08)	2 (4.08)	1.00
Minimal hydrothorax	1 (2.04)	1 (2.04)	1.00
Sepsis	3 (6.12)	5 (10.2)	.73
*Clavien grade III*			
Septic shock	0	1 (2.04)	1.00
Bleeding requiring embolization	0	0	1.00
Bleeding requiring open surgery	0	0	1.00
Postoperative VAS at first day	4.00 ± 0.74	5.00 ± 0.74	.00
Postoperative VAS at third day	2.16 ± 0.47	3.71 ± 0.61	.00
Initial SFR, n/N (%)	39 (79.59)	37 (75.51)	.82
SFR at 1 month, n/N (%)	43 (87.76)	43 (87.76)	1.00

SFR = stone-free rate, VAS = visual analogue score.

## 5. Discussion

Our study found that the 2 surgical methods for octopus stone had the same and higher SFR (87.76% vs 87.76%, *P* = 1.000). Arvind et al reported that multi-channel PCNL has a higher SFR than SM-PCNL combined with RIRS,^[9]^ which was inconsistent with our conclusion. We considered the reason may be as follows: ① each group of operations were conducted by experienced surgeons in our center. ② Compared to a reusable flexible ureteroscope, disposable flexible ureteroscope that we used was better in curvature and could smoothly enter some renal calices with larger angles. ③ It was challenging to deal with stone in the lower calices by flexible ureteroscope, therefore, in combine surgery, we chosen lower calices for the percutaneous renal channel establishing to mark up this shortcoming. ④ In multi-channel PCNL, We increased SFR by increasing the number of channels, especially for stones in parallel renal calices. ⑤ Our center has recently used a ureteral access sheath with a flexible end, combined with negative pressure suction during the operation, which can quickly suck out stone fragments.

Compared to SM-PCNL, MSM-PCNL has a higher blood transfusion rate.^[9]^ However, we found that although the postoperative hemoglobin drop in the multiple channel group was higher than that in the combined group (9.38 ± 7.48 vs 14.22 ± 7.69, *P* = .01), which was considered related to the increase in the number of channels,^[10]^ there was no difference in blood transfusion and selective renal artery embolization rates. This may be due to we giving full play to the flexibility of flexible ureteroscope during combine surgery or increasing the number of channels in MSM-PCNL, insteading the a wide range swing of the nephroscope, and reducing the risk of the calyceal neck and renal parenchyma tear. In addition, the MSM-PCNL group used microchannels instead of the standard channels, and the reduction in channel diameter also effectively reduced the risk of severe bleeding, which was the same conclusion as the study conducted by Güler.^[11]^

**Figure 4.**
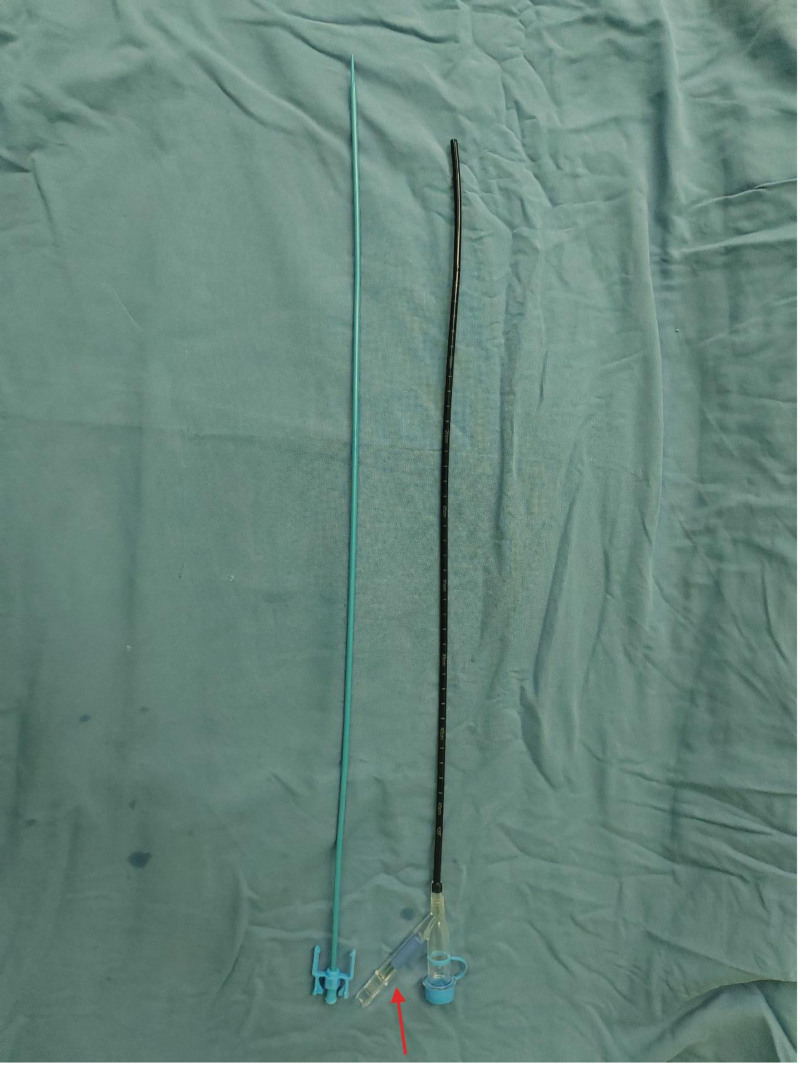
Flexible suction sheath used in combined group, with the red arrow position, was connected to the negative pressure suction device.

There was no significant difference in the fever, sepsis, and septic shock incidence between the 2 groups after the operation. There were no severe organ damage and death cases. Doizi et al found that the pressure in the renal pelvis of S-PCNL was lower than that of simple RIRS and PCNL,^[12]^ and the increase of renal pelvis pressure would also increase the risk of infection.^[13]^ At the same time, microchannels could also increase pressure in the renal pelvis.^[12]^ We paid special attention to this problem in our study during the operation. We communicated the ureteral access sheath with the percutaneous channel to establish a good water circulation in combine surgery. Similarly, multiple percutaneous channels communicated with each other for water circulation in MSM-PCNL. Furthermore, the nephroscope or the ureteral access sheath could also be connected with negative pressure suction device (Fig. 4), which was conducive to maintaining a low renal pelvis pressure, reducing the risk of infection.^[14]^

Removing stone was not our only goal, patients’ comfort should be considered. Postoperative pain seriously affected patients’ experience.^[15]^ In our study, the VAS of the combined group on the 1st and 3rd day after surgery were lower than those of the multiple channel group (4.00 ± 0.74 vs 5.00 ± 0.74, *P* = .00; 2.16 ± 0.47 vs 3.71 ± 0.61, *P* = .00). Zeng et al found that the VAS of the microchannel was lower than that of the standard channel, and the tubeless channel was the most comfortable.^[5]^ The combined group had a higher tubeless rate, and there was no difference in the rates of perirenal hematoma, urine extravasation, and secondary surgery between the 2 groups. For patients without nephrostomy tubes, more attention should be paid to urine color, loin pain, subcutaneous congestion in the waist, and decreased hemoglobin value after surgery. There is no uniform standard for the indications of tubeless, and it is generally believed that there is no planned second operation, no obvious arterial bleeding, no pus, and non-solitary kidney.^[16]^ Our experience suggests that severe hydronephrosis is also a relative contraindication of tubeless because the thin renal parenchyma lacks compression of the puncture channel, which may lead to intractable bleeding if it occured.

However, regarding operation time, the combined group was significantly longer than the multiple-channel group, which was the same as the research conclusion of Zhang et al^[17]^ The analysis reason may be that the efficiency of flexible ureteroscopic lithotripsy and stone extraction was low, especially for patients with long caliceal necks, small infundibulopelvic angle, scattered stone, and high stone CT density. However, the postoperative hospital days of the combined group were less than that of the multiple channel group, which may be related to the less postoperative pain in patients undergoing combined surgery and the faster recovery of patients without nephrostomy tubes.

Our study was retrospective and not randomized and prospective. Therefore, selection bias is difficult to avoid. However, our study used propensity score matching to reduce the impact of related bias and the interference of confounding factors. The study was a single-center retrospective study with limited sample size, which may result in lack of enough confidence on statistical analysis of the data. A multi-center, large-sample, randomized controlled trial will be conducted in the future.

## 6. Conclusion

For octopus stone with a diameter of 2 to 4 cm, the effectiveness and safety of SM-PCNL combined with RIRS were similar to those of MSM-PCNL. The surgeon should carefully evaluate the patient’s physical condition, stone characteristics, and expectations before the operation and assisted the patient in choosing an appropriate plan.

## Author contributions

**Data curation:** Qinghong Ma.

**Methodology:** Jianbin Luo, Guangzhi Wang.

**Project administration:** Guoqiang Chen.

**Writing – original draft:** Deheng Cui.

**Writing – review & editing:** Zesong Yang, Liefu Ye.

## Supplementary Material


